# Complete genome sequence of *Olsenella uli* type strain (VPI D76D-27C^T^)

**DOI:** 10.4056/sigs.1082860

**Published:** 2010-08-20

**Authors:** Markus Göker, Brittany Held, Susan Lucas, Matt Nolan, Montri Yasawong, Tijana Glavina Del Rio, Hope Tice, Jan-Fang Cheng, David Bruce, John C. Detter, Roxanne Tapia, Cliff Han, Lynne Goodwin, Sam Pitluck, Konstantinos Liolios, Natalia Ivanova, Konstantinos Mavromatis, Natalia Mikhailova, Amrita Pati, Amy Chen, Krishna Palaniappan, Miriam Land, Loren Hauser, Yun-Juan Chang, Cynthia D. Jeffries, Manfred Rohde, Johannes Sikorski, Rüdiger Pukall, Tanja Woyke, James Bristow, Jonathan A. Eisen, Victor Markowitz, Philip Hugenholtz, Nikos C. Kyrpides, Hans-Peter Klenk, Alla Lapidus

**Affiliations:** 1 DSMZ - German Collection of Microorganisms and Cell Cultures GmbH, Braunschweig, Germany; 2 DOE Joint Genome Institute, Walnut Creek, California, USA; 3 Los Alamos National Laboratory, Bioscience Division, Los Alamos, New Mexico, USA; 4 Biological Data Management and Technology Center, Lawrence Berkeley National Laboratory, Berkeley, California, USA; 5 Oak Ridge National Laboratory, Oak Ridge, Tennessee, USA; 6 HZI – Helmholtz Centre for Infection Research, Braunschweig, Germany; 7 University of California Davis Genome Center, Davis, California, USA

**Keywords:** microaerotolerant anaerobe, human gingival crevices, primary endodontic infections, *Coriobacteriaceae*, GEBA

## Abstract

*Olsenella uli* (Olsen *et al.* 1991) Dewhirst *et al.* 2001 is the type species of the genus *Olsenella*, which belongs to the actinobacterial family *Coriobacteriaceae*. The species is of interest because it is frequently isolated from dental plaque in periodontitis patients and can cause primary endodontic infection. The species is a Gram-positive, non-motile and non-sporulating bacterium. The strain described in this study was isolated from human gingival crevices. This is the first completed sequence of the genus *Olsenella* and the fifth sequence from  a member of the family *Coriobacteriaceae*. The 2,051,896 bp long genome with its 1,795 protein-coding and 55 RNA genes is a part of the *** G****enomic* *** E****ncyclopedia of* *** B****acteria and* *** A****rchaea * project.

## Introduction

Strain VPI D76D-27C^T^ (= DSM 7084 = ATCC 49627 = JCM 12494) is the type strain of the species *Olsenella uli*, which is the type species of the genus *Olsenella* [1]. Currently, *Olsenella* is one out of thirteen genera in the family *Coriobacteriaceae* [2-4]. This strain was first described in 1991 by Olsen as ‘*Lactobacillus uli*’ [1]. Based on 16S rRNA gene sequence divergence and the presence of unique phenotypic characters, the strain was transferred to the new genus *Olsenella* as *O. uli*. A second novel species, *O. profusa* was also described [5]. More recently, a third species, ‘*O. umbonata*’, was described but is not yet validly published [6].The genus is named in honor of Ingar Olsen, a contemporary Norwegian microbiologist, who first described ‘*L. uli*’. The species epithet ‘uli’ means ‘of the gum’. Periradicular diseases are arguably among the most common human inflammatory diseases [7], which are often the result of microbial infection of the root canal [8]. *Olsenella* species, particularly *O. uli*, are common members of the microbiota associated with primary endodontic infection. *O. uli* has been found to predominate over other Gram-positive rods, (e.g., *Atopobium parvulum*) [9] in root canal samples taken after chemomechanical preparation and intracanal medication, suggesting that this species can resist intracanal disinfection measures and thus may be involved in persistent infections [10,11]. Here we present a summary classification and a set of features for *O. uli* VPI D76D-27C^T^, together with the description of the complete genomic sequencing and annotation.

## Classification and features

Strains from the genus *Olsenella* are found in human oral cavity and likely in bovine rumen [1]. Strain VPI D76D-27C^T^ was isolated from either human gingival crevices or periodontal pockets [1]. The 16S rRNA gene sequence of VPI D76D-27C^T^ is 97% identical to the cultivable strains N13-17 (AY880046) and S13-10 (AY880047). These strains were isolated from a 63-year old male patient with oral squamous-cell carcinoma [12]. Two other uncultured clone sequences with 100% 16S rRNA gene sequence identity to strain VPI D76D-27C^T^ were reported at the Genbank database (status July 2010) [13]. These were clone OPEN_ROOT_17 (FJ982973), isolated from human root canal, and clone BS34 (AY244985) from cow rumen. The 16S rRNA gene of strain VPI D76D-27C^T^ shares 96.7-96.8% sequence identity with the sequences of the type strains from the other members of the genus *Olsenella* [14], whereas the other type strains from the family *Coriobacteriaceae* share 87.3 to 96.7% sequence identity [14]. No phylotypes from environmental screenings or genomic surveys could be linked to the species *O. uli* or to the genus *Olsenella*, indicating a rather rare occurrence of these in the habitats screened thus far (as of July 2010).

**Figure 1:**
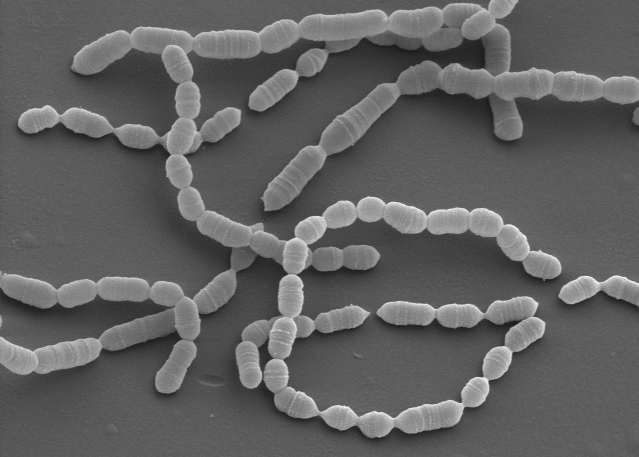
Scanning electron micrograph of *O. uli* VPI D76D-27C^T^

The cells of strain VPI D76D-27C^T^ are nonmotile (Table 1), Gram-positive rods that occur singly, in pairs, and in short chains (Figure 1) [1]. The central part of the cell may swell; particularly when grown on solid medium. Strain VPI D76D-27C^T^ is microaerotolerant to anaerobic [6] with an optimal growth temperature is 37°C [1]. Colonies on brain heart blood agar are one to two mm in diameter, raised or low convex, entire, and translucent to transparent after five days of anaerobic incubation at 37°C [1]. The cells are unable to grow on media with 6.5% w/v of NaCl. Growth is stimulated by Tween 80 (0.02% v/v) . Strain VPI D76D-27C^T^ is able to ferment glycogen, trehalose and starch. Fermentation products from glucose are lactic, acetic, formic and occasionally trace amounts of succinic acid. Lactic acid is a major product of the strain VPI D76D-27C^T^ [1], a feature which was suspected to be important in inflammatory processes of endodontic infections [1,19]. There is little or no gas detected in agar deeps during cell growth. Hydrogen and indole are not produced. Reactions are negative for bile-esculin, DNase and hippurate hydrolysis. This strain does not ferment amygdalin, erythriol, esculin, glycerol, inositol, mannitol, melezitose, melibiose, raffinose, rhamnose, sorbitol and xylose. Acid is produced from fructose, glucose, maltose, mannose, salicin, and sucrose [1]. Strain VPI D76D-27C^T^ does not liquefy gelatin, does not digest meat, does not produce indole, and does not reduce nitrate [1]. It is negative for bile-esculin, DNase, hippurate hydrolysis and catalase activity. However, strain VPI D76D-27C^T^ hydrolyses esculin and produces ammonia from arginine [1].

**Table 1:** Classification and general features of *O. uli* VPI D76D-27C^T^ according to the MIGS recommendations [15].

**MIGS ID**	**Property**	**Term**	**Evidence code**
	Current classification	Domain *Bacteria*	TAS [16]
		Phylum *Actinobacteria*	TAS [17]
		Class *Actinobacteria*	TAS [3,4]
		Subclass *Coriobacteridae*	TAS [3,4]
		Order *Coriobacteriales*	TAS [3,4]
		Suborder *Coriobacterineae*	TAS [3,18]
		Family *Coriobacteriaceae*	TAS [3,4]
		Genus *Olsenella*	TAS [1,5]
		Type strain VPI D76D-27C	TAS [1,5]
	Gram stain	positive	TAS [1]
	Cell shape	small-elliptical rod that occur singly, in pairs or short chains	TAS [1]
	Motility	none	TAS [1]
	Sporulation	none	TAS [1]
	Temperature range	37°C–45°C	NAS
	Optimum temperature	37°C	NAS
	Salinity	< 6.5% NaCl	TAS [1]
MIGS-22	Oxygen requirement	microaerotolerant anaerobic	TAS [6]
	Carbon source	glucose	TAS [1]
	Energy source	chemoorganotroph	TAS [1]
MIGS-6	Habitat	human gingival crevices	TAS [1,5]
MIGS-15	Biotic relationship	free-living	NAS
MIGS-14	Pathogenicity	primary endodontic infections	TAS [11,19,20]
	Biosafety level	2	TAS [21]
	Isolation	plaque from human gingival crevices	TAS [1]
MIGS-4	Geographic location	not reported	
MIGS-5	Sample collection time	1987 or before	TAS [1,5]
MIGS-4.1	Latitude	not reported	
MIGS-4.2	Longitude	not reported	
MIGS-4.3	Depth	not reported	
MIGS-4.4	Altitude	not reported	

Evidence codes - IDA: Inferred from Direct Assay (first time in publication); TAS: Traceable Author Statement (i.e., a direct report exists in the literature); NAS: Non-traceable Author Statement (i.e., not directly observed for the living, isolated sample, but based on a generally accepted property for the species, or anecdotal evidence). These evidence codes are from of the Gene Ontology project [22]. If the evidence code is IDA, then the property was directly observed by one of the authors or an expert mentioned in the acknowledgements

### Chemotaxonomy

Strain VPI D76D-27C^T^ possesses a peptidoglycan type of A4ß based on L-Orn-D-Asp [23]. The major cellular fatty acids of strain VPI D76D-27C^T^ (FAME: fatty acid methyl ester; DMA: dimethylacetyl), when grown in peptone-yeast extract-glucose-Tween 80 broth, are C_18:1_ *cis*9 (32.1%, FAME), C_18:1_ (19.9%, DMA), C_10:0_ (10.1%, FAME), C_17:1_ *cis*8 (6.9%, FAME), C_18:1_ c11/t9/t6 (5.3%, FAME), C_16:1_ *cis9* (4.8%, FAME), C_16:0_ (4.0%, DMA), C_18:1_ *cis*11 (3.6%, DMA), C_14:0_ (3.1%, FAME), C_16:0_ (2.8%, FAME) and C_12:0_ (2.6%, FAME) [1]. More recent data show a somewhat different fatty acid pattern, which is characterized by a large proportion of unbranched fatty acids (C_18:0_, 31-50%) and a quite large proportion of polyunsaturated fatty acids (C_18:2_ cis-9, cis-12; 5.6-8.4%). Presumably, this difference may be attributed to the different growth medium, which is described to be M2 liquid medium containing clarified rumen fluid [6].

**Figure 2:**
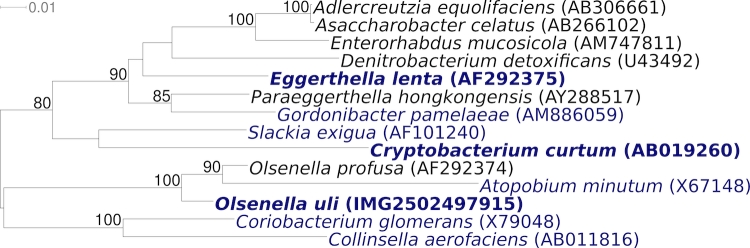
Phylogenetic tree highlighting the position of *O. uli* VPI D76D-27C^T^ relative to the type strains within the genus and the type strains of the other genera within the family *Coriobacteriaceae*. The trees were inferred from 1,408 aligned characters [24,25] of the 16S rRNA gene sequence under the maximum likelihood criterion [26] and as far as possible (note: *Olsenella* is paraphyletic in this tree) rooted in accordance with the current taxonomy [27]. The branches are scaled in terms of the expected number of substitutions per site. Numbers above branches are support values from 250 bootstrap replicates [28] if larger than 60%. Lineages with type strain genome sequencing projects registered in GOLD [29] are shown in blue, published genomes in bold  [30,31]. Adding the 16S rRNA sequence of the type strain of the not yet validly published species ‘*Olsenella umbonata’* (FN178463) to the tree (data not shown) did not change the overall arrangement; ‘*O. umbonata’* appeared within the grade between *O. uli* and *O. profusa*.

Figure 2 shows the phylogenetic neighborhood of *O. uli* VPI D76D-27C^T^ in a 16S rRNA based tree. The sequence of the unique 16S rRNA gene is identical with the previously published sequence generated from ATCC 49627 (AF292373).

## Genome sequencing and annotation

### Genome project history

This organism was selected for sequencing on the basis of its phylogenetic position [32], and is part of the *** G****enomic* *** E****ncyclopedia of* *** B****acteria and* *** A****rchaea * project [33]. The genome project is deposited in the Genome OnLine Database [29] and the complete genome sequence is deposited in GenBank. Sequencing, finishing and annotation were performed by the DOE Joint Genome Institute (JGI). A summary of the project information is shown in Table 2.

**Table 2:** Genome sequencing project information

**MIGS ID**	**Property**	**Term**
MIGS-31	Finishing quality	Finished
MIGS-28	Libraries used	Three genomic libraries: one 454 standard and one 454 10 kb pyrosequence library; one Illumina library
MIGS-29	Sequencing platforms	454 GS Titanium, Illumina GAii
MIGS-31.2	Sequencing coverage	90.5× pyrosequence, 211.8× Illumina
MIGS-30	Assemblers	Newbler version 2.0.1- PreRelease-03-30-2009-gcc-3.4.6-threads, phrap, Velvet
MIGS-32	Gene calling method	Prodigal 1.4, GenePRIMP
	INSDC ID	CP002106
	Genbank Date of Release	August 6, 2010
	GOLD ID	Gc01331
	NCBI project ID	36641
	Database: IMG-GEBA	2502422319
MIGS-13	Source material identifier	DSM 7084
	Project relevance	Tree of Life, GEBA

### Growth conditions and DNA isolation

*O. uli* VPI D76D-27C^T^, DSM 3776, was grown in medium 104 (PYG modified medium) [34] at 37°C under strictly anaerobic conditions. DNA was isolated from 0.5-1 g of cell paste using MasterPure Gram Positive DNA Purification Kit (Epicentre MGP04100) following the standard protocol as recommended by the manufacturer, with modification st/LALM for cell lysis as described in [33].

### Genome sequencing and assembly

The genome of *O. uli* was sequenced using a combination of Illumina and 454 technologies. An Illumina GAii shotgun library with reads of 434,617,748 bp, a 454 Titanium draft library with average read length of 302 +/- 162.3 bp bases, and a paired end 454 library with average insert size of 9.273 +/- 2.318 kb were generated. All general aspects of library construction and sequencing can be found at http://www.jgi.doe.gov/. Illumina sequencing data was assembled with VELVET, and the consensus sequences were shredded into 1.5 kb overlapped fake reads and assembled together with the 454 data. Draft assemblies were based on 2.0 Mb 454 draft data, and 454 paired end data. Newbler parameters are -consed -a 50 -l 350 -g -m -ml 20. The initial Newbler assembly contained 149 contigs in 1 scaffold. We converted the initial 454 assembly into a phrap assembly by making fake reads from the consensus, collecting the read pairs in the 454 paired end library. The Phred/Phrap/Consed software package (www.phrap.com) was used for sequence assembly and quality assessment [35] in the following finishing process. After the shotgun stage, reads were assembled with parallel phrap (High Performance Software, LLC). Possible mis-assemblies were corrected with gapResolution (http://www.jgi.doe.gov/), Dupfinisher [35], or sequencing cloned bridging PCR fragments with subcloning or transposon bombing (Epicentre Biotechnologies, Madison, WI). Gaps between contigs were closed by editing in Consed, by PCR and by Bubble PCR primer walks (J.-F. Chang, unpublished). A total of 394 additional Sanger reactions were necessary to close gaps and to raise the quality of the finished sequence. Illumina reads were also used to improve the final consensus quality using an in-house developed tool (the Polisher [36], ). The error rate of the final genome sequence is less than 1 in 100,000.

### Genome annotation

Genes were identified using Prodigal [37] as part of the Oak Ridge National Laboratory genome annotation pipeline, followed by a round of manual curation using the JGI GenePRIMP pipeline [38]. The predicted CDSs were translated and used to search the National Center for Biotechnology Information (NCBI) nonredundant database, UniProt, TIGRFam, Pfam, PRIAM, KEGG, COG, and InterPro databases. Additional gene prediction analysis and functional annotation was performed within the Integrated Microbial Genomes - Expert Review (IMG-ER) platform [39].

## Genome properties

**Figure 3:**
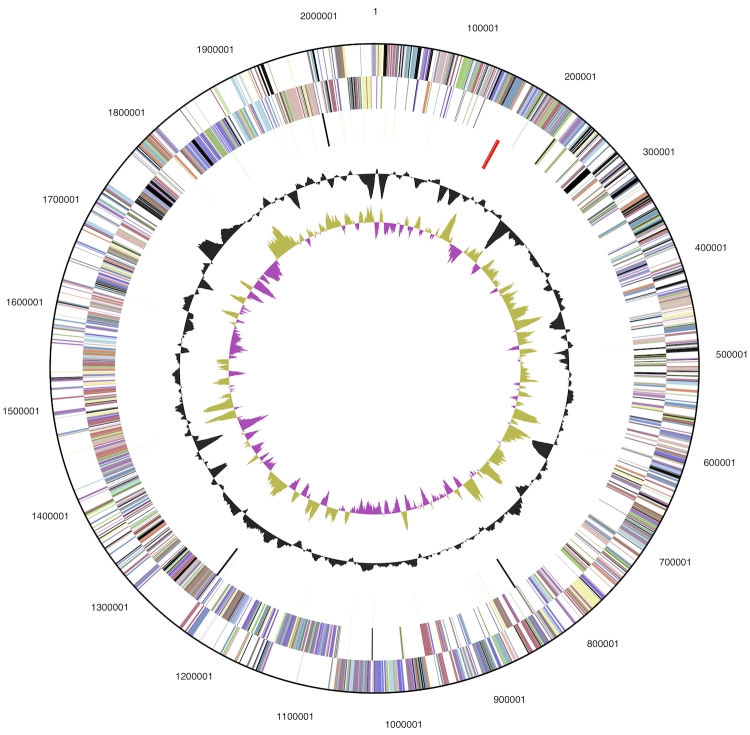
Graphical circular map of the genome. From outside to the center: Genes on forward strand (color by COG categories), Genes on reverse strand (color by COG categories), RNA genes (tRNAs green, rRNAs red, other RNAs black), GC content, GC skew.

The genome consists of a 2,051,896 bp long chromosome with a 64.7% GC content (Table 3 and Figure 3). Of the 1,850 genes predicted, 1,795 were protein-coding genes, and 55 RNAs; fifty six pseudogenes were also identified. The majority of the protein-coding genes (75.9%) were assigned a putative function while the remaining ones were annotated as hypothetical proteins. The distribution of genes into COGs functional categories is presented in Table 4.

**Table 3:** Genome Statistics

**Attribute**	**Value**	**% of Total**
Genome size (bp)	2,051,896	100.00%
DNA coding region (bp)	1,789,074	87.19%
DNA G+C content (bp)	1,327,526	64.70%
Number of replicons	1	
Extrachromosomal elements	0	
Total genes	1,850	100.00%
RNA genes	55	2.97%
rRNA operons	1	
Protein-coding genes	1,795	97.03%
Pseudo genes	56	3.03%
Genes with function prediction	1,404	75.89%
Genes in paralog clusters	160	8.65%
Genes assigned to COGs	1,412	76.32%
Genes assigned Pfam domains	1,429	77.24%
Genes with signal peptides	320	17.30%
Genes with transmembrane helices	423	22.86%
CRISPR repeats	1	

**Table 4:** Number of genes associated with the general COG functional categories

**Code**	**value**	**%age**	**Description**
J	137	8.9	Translation, ribosomal structure and biogenesis
A	0	0.0	RNA processing and modification
K	140	9.1	Transcription
L	82	5.3	Replication, recombination and repair
B	1	0.1	Chromatin structure and dynamics
D	17	1.1	Cell cycle control, cell division, chromosome partitioning
Y	0	0.0	Nuclear structure
V	44	2.9	Defense mechanisms
T	72	4.7	Signal transduction mechanisms
M	86	5.6	Cell wall/membrane/envelope biogenesis
N	0	0.0	Cell motility
Z	0	0.0	Cytoskeleton
W	0	0.0	Extracellular structures
U	18	1.2	Intracellular trafficking, secretion, and vesicular transport
O	51	3.3	Posttranslational modification, protein turnover, chaperones
C	75	4.9	Energy production and conversion
G	165	10.7	Carbohydrate transport and metabolism
E	150	9.7	Amino acid transport and metabolism
F	56	3.6	Nucleotide transport and metabolism
H	46	3.0	Coenzyme transport and metabolism
I	32	2.1	Lipid transport and metabolism
P	66	4.3	Inorganic ion transport and metabolism
Q	24	1.6	Secondary metabolites biosynthesis, transport and catabolism
R	172	11.1	General function prediction only
S	112	7.2	Function unknown
-	438	23.7	Not in COGs
